# Supplementary material for: The Effects of Physiotherapy in the Treatment of Cubital Tunnel Syndrome: A Systematic Review
Source: J Clin Med. 2022 Jul 21;11(14):4247. doi: 10.3390/jcm11144247 (PMC9318553; doi:10.3390/jcm11144247)
Supplement: Supplementary file 1 [file jcm-11-04247-s001.zip › jcm-1830390-supplementary.pdf]

Search strategy

#1 cubital tunnel syndrome
#2 cubital tunnel
#3 ulnar nerve
#4 ulnar neuropathy
#5 entrapment neuropathy
#6 Ulnar Nerve Compression Syndromes cubital tunnel syndrome[MeSH Terms]
#7 Ulnar Nerve Compression Syndromes[MeSH Terms]
#8 #1 OR #2 OR #3 OR #4 OR #5 OR #6 OR #7
#9 physiotherapy
#10 physical therapy
#11 manual therapy
#12 joint manipulation
#13 joint mobilisation
#14 neurodynamic techniques
#15 neurodynamic mobilization
#16 neural mobilization
#17 nerve-gliding techniques
#18 nerve mobilization
#19 exercises
#20 electrotherapy
#21 electrophysiological modalities
#22 magnetotherapy
#23 dry needling
#24 laser therapy
#25 ultrasound
#26 cryotherapy
#27 cold therapy
#28 shock wave therapy
#29 #9 OR #10 OR #11 OR #12 OR #13 OR #14 OR #15 OR #16 OR #17 OR #18 OR #19 OR #20 OR #21 OR #22 OR #23 OR #24 OR #25 OR #26 OR #27 OR#28
#30 case study
#31 case report
#32 placebo
#33 sham
#34 randomized control trial
#35 randomised control trial
#36 randomized clinical trial
#37 randomised clinical trial)
#38 #30 OR #31 OR #32 OR #33 OR #34 OR #35 OR #36 OR #37
#39 nerve conduction study
#40 electrodiagnostic test

#41 sensory threshold
#42 sensation test
#43 two-point discrimination
#44 cutaneous pressure threshold
#45 vibration sensation
#46 muscle strength
#47 grip strength
#48 pinch strength
#49 pain measurement
#50 the numeric pain rating scale
#51 visual analoge scale
#52 functional tests
#53 patient-specific functional scale
#54 global rating of change
#55 northwick park questionnaire
#56 disability of arm shoulder
#57 hand index
#58 phalen’s test
#59 tinel’s sign
#60 froment sign
#61 elbow flexion test
#62 ulnar nerve provocation test
#63 nerve provocation test
#64 range of motion
#65 upper limb neurodynamic test
#66 electromyography
#67 #39 OR #40 OR #41 OR #42 OR #43 OR #44 OR #45 OR #46 OR #47 OR #48 OR #49 OR #50 OR #51 OR #52 OR #53 OR #54 OR #55 OR #56 OR #57 OR #58 OR #59 OR #60 OR #61 OR #62 OR #63 OR #64 OR #65 OR #66
#68 #8 AND #29 AND #38 AND #67
